# Current indications for post-mastectomy radiation

**DOI:** 10.1186/1477-7800-6-5

**Published:** 2009-02-09

**Authors:** Maria Vilarino-Varela, Yaw Sinn Chin, Andreas Makris

**Affiliations:** 1 Academic Oncology Unit, Mount Vernon Cancer Centre, Rickmansworth Road, Northwood, Middlesex, HA6 2RN, UK; 2 Cancer Care Centre, St. George Hospital, Short Street, Kogarah, NSW 2217, Australia

## Abstract

It has been long established that post-mastectomy radiotherapy reduces the risk of locoregional failure. A survival advantage, however, has only recently been demonstrated. We here provide a review of the literature as regards to the current indications for post-mastectomy radiotherapy.

## Current indications for post-mastectomy radiation

Primary breast carcinoma may be managed surgically with either mastectomy or breast conserving surgery. Although radiotherapy following breast conserving surgery is widely accepted, the role of post-mastectomy radiotherapy (PMRT) has been a subject for debate for many years. PMRT has been known since the 1970's to substantially reduce the risk of locoregional failure (LRF), however, a disease specific survival and overall survival advantage has only more recently been demonstrated [1-5]. The systemic therapy of breast cancer has also evolved since many of the studies were conducted, raising the issue of how best to incorporate PMRT into current clinical practice.

Randomized control studies of PMRT have consistently reported improved rates of locoregional control by two-thirds regardless of patient characteristics such as age, treatment era or disease characteristics [5-7]. Only in the late 1990's was it noted that PMRT conveyed a survival advantage in high risk patients treated with adjuvant chemotherapy or hormonal therapy [1-5]. The Danish 82b study of PMRT in premenopausal women receiving adjuvant CMF chemotherapy reported a reduction in LRF (9% v. 32%, p < 0.001) and improved 10 year overall survival (54% v. 45%, p < 0.001) in patients receiving PMRT [2] with similar results reported in the British Columbia study [1]. The Danish 82c study assessing PMRT in postmenopausal women receiving one year adjuvant tamoxifen also revealed similar reductions in LRF (8% v. 35%, p < 0.001) and improved 10 year overall survival (45% v. 36%, p = 0.03) [3].

There are a number of reasons why a survival advantage may not have initially been apparent. Original studies were conducted in the era prior to adjuvant systemic therapy. The resulting reduction of distant failure with systemic therapy may allow the survival effect of PMRT to be more evident. Analysis of cause-specific mortality in the older PMRT studies revealed that the reduction in breast cancer deaths was cancelled out by an increase in late cardiac mortality secondary to radiotherapy [6,7]. With modern radiotherapy techniques delivery of dose to the chest wall is more uniform and cardiac dose minimized. Older studies were also not stratified according to risk and being of small sample size not powered sufficiently to detect a small survival advantage.

The absolute benefit gained from PMRT is believed greatest for those at high risk of LRF. There is consensus that PMRT should be considered when risk of LRF is greater than 20%, such as for patients with four or more positive axillary lymph nodes, primary tumour size 5 cm or more, T4 disease and positive/very close margin [8-11]. PMRT is not indicated in patients with tumours less than 5 cm in size and negative axillary nodes as there is only small benefit in terms of locoregional control, and insignificant absolute survival advantage. Several studies have suggested other factors that may contribute to risk of LRF particularly when present in combination [8-11]. These include age less than 40 years, histological grade 3 tumours, presence of lymphovascular invasion, less than 6 nodes removed at axillary dissection and significant nodal extracapsular spread (> 2 mm). The merit of PMRT, however, in this group of patients is not known.

It remains unclear whether patients with one to three axillary nodes positive benefit significantly from PMRT. A number of collaborative groups (including the Eastern Cooperative Oncology Group, the International Breast Cancer Study Group and the National Surgical Adjuvant Breast and Bowel Project) have reported patterns of LRF following mastectomy in a number of their trials and have reported that this group of patients experience a LRF rate of 13–19% compared with 30–33% as reported in the key randomised trials [8-11]. This discrepancy may be explained by limited axillary dissection underestimating the true extent of axillary nodal involvement as only a median of 7 axillary nodes were removed in the Danish study and 11 nodes in the British Columbia study compared with 15–17 nodes in these other studies (which were not directly investigating the value of PMRT). A subset analysis of the Danish 82 b and c studies has been performed including only those with 8 or more axillary nodes removed reporting continued significant and equal reductions in LRF and overall survival at 15 years with PMRT in both the one to three and greater than four involved node groups[4]. LRF, however, remained high in the subgroup analysed and the caveat remains that the extent of adjuvant systemic therapy received in the Danish studies was less than current clinical practice which may affect the perceived benefit of PMRT in this group. To resolve whether patients with one to three positive axillary nodes should undergo PMRT a phase III randomised control trial – the Selective Use of Post-operative Radiotherapy after Mastectomy (SUPREMO) trial – is currently being conducted in Europe randomising patients with tumours less than 5 cm in size and 0–3 positive axillary nodes.

The commonest site of LRF following mastectomy and axillary nodal dissection is the chest wall (50–75%) followed by the supraclavicular fossa and infraclavicular region (20–40%) [8,9]. The rate of axillary recurrence following a level I/II axillary dissection is < 5% at 10 years [12,13] and as such the axilla is not routinely irradiated as part of PMRT. Furthermore, irradiating the entire axilla following axillary dissection would significantly increase the rate of chronic arm morbidity and lymphoedema [14,15].

Irradiation of the internal mammary nodal region remains controversial. Clinical evidence of recurrence at this site appears uncommon [8-11] despite older surgical reports suggesting high rates of involvement [16,17]. The Danish and British Columbia studies incorporated internal mammary nodal irradiation [1-3] but its contribution to survival is unclear. There remains a particular concern regarding radiation dose to the heart from treatment of the internal mammary chain and thereby increased risk of late cardiac toxicity. Results from the European Organisation for Research and Treatment of Cancer (EORTC) 22922 and the National Cancer Institute of Canada Clinical Trials Group (NCIC CTG) MA20 randomised trials may provide further clarification.

Systemic therapy of early breast cancer has evolved considerably since many of the PMRT studies were conducted. Women are more likely to receive more cardiotoxic adjuvant systemic therapy. There is a significant survival advantage to anthracyline based regimens compared to CMF [18], and as a result anthracyclines are now routinely incorporated into most adjuvant chemotherapy schedules. High risk patients may also receive taxanes following evidence of a further survival advantage when added to anthracyclines [19,20]. Furthermore, recent studies have demonstrated a survival benefit from the addition of trastuzumab in patients with HER2-positive tumours [21,22]. The potential cardiotoxicity of trastuzumab is now well-established. Whether PMRT offers a further advantage in patients receiving optimal systemic adjuvant therapy is unclear. However, the MD Anderson Cancer Center and the NSABP series assessing locoregional recurrence in patients receiving anthracycline based chemotherapy suggest that locoregional failure remains an important concern [9,11]. The current recommended duration of adjuvant hormonal therapy is 5 years, compared to one as in the Danish 82c study, which may also have an impact on LRF. Furthermore, in postmenopausal women, the aromatase inhibitors (AIs) are increasingly being used instead of tamoxifen. AIs have been shown to reduce both local and distal relapse compared to tamoxifen [23-26].

## Conclusion

PMRT reduces the risk of LRF and increases overall survival in high risk patients. Current indications for PMRT include axillary nodal involvement of 4 or more nodes, disease 5 cm or more in size, T4 disease and positive surgical margins. The potential benefits of PMRT in patients with < 4 positive axillary nodes remain controversial and recruitment to ongoing clinical trials should be encouraged.

## Competing interests

The authors declare that they have no competing interests.

## Authors' contributions

MVV performed literature search, reviewed literature and drafted manuscript. YSC assisted literature search and in drafting of manuscript. AM proposed the review, revised manuscript critically for intellectual content. All authors read and approved the final manuscript
